# Aerobic Training after Myocardial Infarction: Remodeling Evaluated by Cardiac Magnetic Resonance

**DOI:** 10.5935/abc.20160031

**Published:** 2016-04

**Authors:** Nataly Lino Izeli, Aurélia Juliana dos Santos, Júlio César Crescêncio, Ana Clara Campagnolo Real Gonçalves, Valéria Papa, Fabiana Marques, Antônio Pazin-Filho, Lourenço Gallo-Júnior, André Schmidt

**Affiliations:** 1 Divisão de Cardiologia do Hospital das Clínicas da Faculdade de Medicina de Ribeirão Preto - USP, Ribeirão Preto, SP - Brazil; 2 Divisão de Emergência da Faculdade de Medicina de Ribeirão Preto - USP, Ribeirão Preto, SP - Brazil

**Keywords:** Exercise, Rehabilitation, Myocardial Infarction, Magnetic Resonance Spectroscopy

## Abstract

**Background:**

Numerous studies show the benefits of exercise training after myocardial infarction (MI). Nevertheless, the effects on function and remodeling are still controversial.

**Objectives:**

To evaluate, in patients after (MI), the effects of aerobic exercise of moderate intensity on ventricular remodeling by cardiac magnetic resonance imaging (CMR).

**Methods:**

26 male patients, 52.9 ± 7.9 years, after a first MI, were assigned to groups: trained group (TG), 18; and control group (CG), 8. The TG performed supervised aerobic exercise on treadmill twice a week, and unsupervised sessions on 2 additional days per week, for at least 3 months. Laboratory tests, anthropometric measurements, resting heart rate (HR), exercise test, and CMR were conducted at baseline and follow-up.

**Results:**

The TG showed a 10.8% reduction in fasting blood glucose (p = 0.01), and a 7.3-bpm reduction in resting HR in both sitting and supine positions (p < 0.0001). There was an increase in oxygen uptake only in the TG (35.4 ± 8.1 to 49.1 ± 9.6 mL/kg/min, p < 0.0001). There was a statistically significant decrease in the TG left ventricular mass (LVmass) (128.7 ± 38.9 to 117.2 ± 27.2 g, p = 0.0032). There were no statistically significant changes in the values of left ventricular end-diastolic volume (LVEDV) and ejection fraction in the groups. The LVmass/EDV ratio demonstrated a statistically significant positive remodeling in the TG (p = 0.015).

**Conclusions:**

Aerobic exercise of moderate intensity improved physical capacity and other cardiovascular variables. A positive remodeling was identified in the TG, where a left ventricular diastolic dimension increase was associated with LVmass reduction.

## Introduction

Left ventricular (LV) remodeling after myocardial infarction (MI) is a complex and multifactorial process with prognostic and therapeutic implications.^1^ Minimizing LV remodeling with medications improved survival and quality of life.^2-4^

Exercise training has been shown to improve exercise capacity and reduce mortality, amplifying potential therapeutic interventions.^5^ In addition, aerobic exercise reduces cardiovascular risk factors, which makes it further appealing as an adjuvant treatment.^6,7^

The benefits of exercise training in increasing aerobic capacity, as well as other hemodynamic changes, are well documented. Nevertheless, the effects of exercise on myocardial function and LV remodeling after MI are still controversial. Some studies have suggested that exercise after MI further deteriorates cardiac function due to additional stress over the infarcted area, infarct expansion, aneurysm formation, or ejection fraction (EF) reduction.^8-10^ Numerous studies have failed to confirm these findings and suggested that exercise does not alter ventricular parameters even in different training intensities.^11-14^ Other studies have shown that after a recent acute MI with systolic dysfunction, exercise can attenuate ventricular remodeling and even reverse this process.^15-18^

Heterogeneity related to patient sampling, intensity of training, measurement techniques, or even a combination of these factors could be potential explanations for that divergence.^19^

We sought to evaluate the effects of aerobic exercise of moderate intensity, performed in patients after MI on cardiac function through cardiac magnetic resonance (CMR), a well-recognized gold standard technique for the quantification of ventricular volumes, EF and myocardial mass.^20^

## Methods

Male patients were selected according to strict inclusion criteria during an 18-month period. All patients presented an acute MI with ST elevation. Patients enrolled should be younger than 70 years, clinically stable, on sinus rhythm and not previously included in any cardiac rehabilitation program. It must also be no later than 6 months after their first MI. Exclusion criteria included unstable coronary artery disease, uncontrolled hypertension, malignant ventricular arrhythmia and ventricular failure during exercise, Chagas disease, untreated thyroid function disorders, neurological or orthopedic inability to perform physical exercises on a treadmill, and general debility. Since cardiac rehabilitation was offered to all patients as part of our institution standard of care, patients with inclusion criteria but not willing to engage in the program, who accepted to perform the exams of the protocol, were included as controls.

The study was approved by the local ethical committee. Written informed consent was obtained from all patients.

### Study Design

Initially, all patients were evaluated by a cardiologist to provide a clinical history and undergo physical examination with anthropometric measurements. When necessary, medications were optimized. Patients in both groups underwent laboratory tests that included complete blood count, lipid profile, and fasting glucose. A functional evaluation was performed at baseline and after at least 3 months of aerobic exercise training, including resting heart rate (HR), exercise testing and CMR. The first two were always performed in the morning, and no medication was withdrawn. The intervention period (IP) for the trained group (TG) was defined as the time between the first exercise training session and the final CMR and clinical and laboratorial evaluations. The IP for the control group (CG) was the period between the two CMR exams.

### Resting HR

The resting HR was obtained beat-by-beat, using a modified MC5 electrocardiogram lead, in the morning, and on currently prescribed medications. The volunteers remained at rest in the supine position for 15 minutes, and in the sitting position for 8 minutes. Patients were instructed to maintain a relaxed posture without moving arms and legs, talking or sleeping. To obtain the mean HR, the first ten beats were discarded and arithmetic mean was performed with the other values.

### Exercise Testing

Patients performed a symptom-limited treadmill exercise test with electrocardiographic monitoring of three leads (MC5, D2M, and V2M), using the *Micromed Ergo PC* software (São Paulo, Brazil). They were instructed not to consume stimulating food substances, and not to perform strenuous activities before the test. They underwent the modified Balke protocol, with increments in treadmill speed and inclination every minute, selected according to the physical capacity expected for each patient. The electrocardiogram was monitored continuously. Blood pressure, HR, and signs and symptoms were obtained every minute during exercise, and throughout the recovery period. The following variables were obtained at peak exercise: HR, blood pressure, oxygen uptake, metabolic equivalent, treadmill load, and rating of perceived exertion (Borg CR10 scale).^21^ The peak oxygen uptake was obtained indirectly, using the treadmill speed and inclination at peak exercise.

### MRI

All imaging was performed using a 1.5T unit (Magneton Vision, Siemens, Erlängen, Germany). After initial scout imaging, breath-hold steady-state free precession cine MR images were acquired along the vertical long axis (2- and 4-chamber view) and a short axis stack (contiguous 8-mm-thick slices) covering the ventricle extension. The later sequence was used to assess the LV mass (LVmass), LV dimensions and EF. All images were blindly analyzed by a single operator (A.S.) using Image J.^22^ LV end-systolic volume and LV end-diastolic volume (LVEDV) were calculated using Simpson's rule. The LVmass was determined by the sum of the myocardial area (LV epicardial contour minus LV endocardial contour) times slice thickness, and multiplied by the specific myocardial gravity (1.05g/mL). The LVEF was calculated as the difference between LVEDV and LV end-systolic volume, divided by LVEDV and multiplied by 100. No gadolinium infusion was used.

### Exercise Training Protocol

The aerobic exercise training was prescribed based on the peak HR or HR in the ischemic threshold obtained during exercise testing. Exercise intensity was determined at 50-70% of HR reserve (Karvonen's equation). The TG patients participated in a supervised 30-minute treadmill session, twice a week in the morning period, for at least 3 months. Each session was preceded by a 5-minute warm-up and followed by a 5-minute cooling-down period. During each supervised session, intensity of exercise (treadmill speed and inclination), HR, blood pressure, and rating of perceived exertion (Borg CR10 scale) were recorded. Patients were instructed to undergo more two unsupervised sessions each week, adjusting the speed of walking by counting the radial pulse or using a pulse HR monitor. Data from outside walking were registered in a dairy, in which the patient reported the resting HR, exercise time, and HR during walking and after 5 minutes of recovery. During training sessions, the TG patients received information regarding lifestyle modification strategies, regular physical activity, healthy diet, importance of weight control, and stress reduction. The CG underwent the usual clinical follow-up and was subsequently contacted to perform the final exams. The volunteers' medications were not modified during the IP.

### Statistical Analysis

Continuous variables were expressed as mean ± standard deviation. Categorical variables were presented as percentages. The distribution of the data was analyzed with the Shapiro-Wilk test. Categorical data were compared with the chi-square and Fisher exact tests. Continuous data were assessed by the Wilcoxon nonparametric rank-sum test (intragroup analysis) and Mann-Whitney nonparametric test (intergroup analysis), with a significance level of 5%. Statistical analysis was performed using the SPSS for Windows software (version 10.0, SPSS Inc., Chicago Illinois, USA).

## Results

A total of 26 male patients (52.9 ± 7.9 years) were enrolled in the study after fulfilling the inclusion criteria and presenting no exclusion criteria. Since 8 of them were not willing to participate in the rehabilitation program, but agreed to undergo the tests needed, they constituted the CG. The other 18 patients were the TG. Seventeen patients received fibrinolytics on MI admission (TG = 10 and CG = 7; p = 0.29). The TG had a lower prevalence of smoking and sedentary lifestyle than the CG. Baseline clinical data of the two groups are summarized in Table 1. The IP was 136.7 ± 26.2 days for the TG, and 150.5 ± 44.5 days for the CG (p = 0.87). The TG performed a mean of 27.5 ± 5.6 supervised training sessions. None of the groups had clinical complications during the IP.

**Table 1:** Baseline characteristics of the trained and control groups (TG and CG, respectively)

	**TG n = 16**	**CG n = 8**	**p value**
Age (years)	54.1 ± 7.0	50.3 ± 9.7	0.87
Time from MI (days)	145.0 ± 104.7	117.5 ± 91.1	0.99
Killip class, I/II/III (n)	10/8/0	4/3/1	0.31
Weight (kg)	80.0 ± 14.8	90.5 ± 12.4	0.08
Body mass index (kg/m^2^)	28.1 ± 3.9	30.2 ± 3.1	0.34
EF (%)	45.1 ± 11.8	44.9 ± 11.0	0.80
Culprit lesion artery (%)			0.60
Left anterior descending	66.6	50.0	
Left circumflex coronary	16.7	37.5	
Right coronary artery	16.7	12.5	
Revascularization procedures (n)			0.44
PTCA	14	5	
CABG surgery	1	0	
**Cardiovascular risk factors (%)**			
Hypertension	61.1	75.0	0.67
Dyslipidemia	66.7	37.5	0.22
Diabetes mellitus	22.2	37.5	0.64
Family history	44.4	37.5	1.00
Current smokers	0	50.0	0.0047
Sedentary	50.0	100	0.0098
Overweight	72.2	100	0.10
**Medical therapy (%)**			
Antithrombotic agent	100	100	1.00
β-blocker	100	100	1.00
ACEI or ARB	88.8	87.5	1.00
Statin	100	100	1.00
Diuretics	22.2	25.0	1.00

MI: myocardial infarction; EF: ejection fraction; PTCA: percutaneous transluminal coronary angioplasty; CABG: coronary artery bypass grafting; ACEI: angiotensin-converting-enzyme inhibitors; ARB: angiotensin-receptor blockers.

### Anthropometric measurements and laboratory tests

Baseline anthropometric measurements were similar between the two groups. At the end of the IP, the TG showed a decrease of 1.28 kg in weight and of 0.47 kg/m^2^ in body mass index (BMI), with no statistical difference (p = 0.17 and p = 0.15, respectively). We observed a statistically significant increase in weight (3.8kg) and BMI (1.27 kg/m^2^) in the CG (p = 0.04 for both).

There were no differences between the groups for baseline measures of total cholesterol (p = 0.64), triglycerides (p = 0.19), high-density-lipoprotein cholesterol (HDL-c) (p = 0.4530), low-density-lipoprotein cholesterol (LDL-c) (p = 0.53) and fasting glucose (p = 0.52) (Table 2). The TG showed changes in lipids and glucose levels at the end of the exercise training protocol. Fasting glucose decreased significantly (106.0 ± 26.4 to 94.5 ± 14.8 mg/dL, p = 0.01). The lipid profile showed improvement without statistical significance. Total cholesterol was reduced by 6% (p = 0.08). There was a mean reduction of 16.9% in triglycerides (p = 0.14). HDL-c increased 5.1% (p = 0.42), LDL-c decreased 6.1% (p = 0.32). In CG there was a trend to increase in total cholesterol (p = 0.46), triglycerides (p = 0.11) and fasting glucose (p = 0.47).

**Table 2:** Lipid profile and fasting glucose values (mean ± SD) in the trained and control groups (TG and CG, respectively) at baseline and follow-up

**Laboratory tests**	**TG**		**CG**	
	**Baseline**	**Follow-up**	**Baseline**	**Follow-up**
Total cholesterol (mg/dL)	157.4 ± 43.8	147.9 ± 47.9	170.1 ± 44.8	178.1 ± 44.5
Triglycerides (mg/dL)	168.5 ± 76.7	140.1 ± 66.4	226.0 ± 112.9	319.1 ± 194.1
HDL-c (mg/dL)	37.4 ± 7.1	39.3 ± 10.0	39.8 ± 4.1	40.4 ± 5.7
LDL-c (mg/dL)	86.3 ± 36.8	81.0 ± 34.8	73.8 ± 12.2	73.2 ± 27.3
Fasting glucose (mg/dL)	106.0 ± 26.4	94.5 ± 14.8*	114.8 ± 42.5	122.4 ± 38.8

* p = 0.011.

### Resting HR

No statistically significant difference was found at baseline between groups in resting HR. The resting HR in TG in the sitting position showed a decrease from 62.4 ± 9.1 to 55.1 ± 5.9 bpm (p < 0.0001). In the supine position, HR decreased from 61.6 ± 9.7 to 54.3 ± 6.5 bpm (p < 0.0001). No changes were observed in the CG.

### Exercise testing

Chest pain was the reason for interruption in 3 participants of the TG at baseline, and 2 had it again in the second exercise test. No CG participant had chest pain in the baseline exam, but it was the reason for interruption of one participant in the second test. Data from the exercise tests are summarized in Table 3. No differences were observed within or between groups in maximal HR or systolic blood pressure at baseline and follow-up. The TG demonstrated a 38.7% increase in maximal oxygen uptake (p < 0.0001). At the end of the training protocol, a statistically significant increase in the maximum treadmill load, expressed by values of speed and inclination in the TG, was noted. No changes occurred in the CG.

**Table 3:** Data from exercise tests (mean ± SD) from the trained and control groups (TG and CG, respectively) at baseline and follow-up

**Exercise tests**	**TG**		**CG**	
	**Baseline**	**Follow-up**	**Baseline**	**Follow-up**
Peak HR (bpm)	132.0 ± 20.2	140.2 ± 20.1	127.0 ± 21.0	125.4 ± 26.5
Peak systolic blood pressure (mmHg)	178.3 ± 24.6	181.1 ± 22.1	183.8 ± 16.0	193.8 ± 23.4
Peak HR-pressure product (bpm.mmHg)	23598.6 ± 5093.5	25540.8 ± 5640.0	23612.5 ± 6353.0	24312.5 ± 5997.9
Metabolic equivalent (MET)	10.1 ± 2.3	14.0 ± 2.8*	8.7 ± 2.7	8.6 ± 2.5
Peak oxygen uptake (mL/kg/min)	35.4 ± 8.1	49.1 ± 9.6*	30.3 ± 9.5	30.2 ± 8.9
Speed (mph)	3.07 ± 0.5	3.8 ± 0.7*	2.9 ± 0.3	2.9 ± 0.5
Ramp inclination (%)	15.9 ± 3.6	19.1 ± 3.0#	13.5 ± 4.9	13.0 ± 3.5

HR: heart rate.

* p < 0.0001;

# p = 0.0026.

### MRI

Table 4 shows the LVEDV, LVmass, EF values, and LVmass/EDV ratio in both groups. Baseline LV parameters were similar in the two groups, except for ventricular mass (p = 0.0225) and indexed LVmass (p = 0.0429), which were higher in the CG. The LVEDV increased slightly in both groups, without significant differences. The EF showed no significant modification in the groups during the study.

**Table 4:** Cardiac magnetic resonance (CMR) measurements (mean ± SD) from the trained and control groups (TG and CG, respectively) at baseline and follow-up

**CMR**	**TG**		**CG**	
	**Baseline**	**Follow-Up**	**Baseline**	**Follow-Up**
EDV (mL)	110.7 ± 43.5	116.8 ± 38.2	126.3 ± 39.4	134.3 ± 42.2
Indexed EDV (mL/m^2^)	38.5 ± 14.1	40.6 ± 12.3	42.2 ± 12.5	44.4 ± 11.9
EF (%)	45.1 ± 11.8	46.8 ± 10.0	44.9 ± 11.0	42.6 ± 11.6
LVmass (g)	128.7 ± 38.9	117.2 ± 27.2*	159.6 ± 29.3#	167.8 ± 49.7
Indexed LVmass (g/m^2^)	44.9 ± 12.5	40.9 ± 8.6*	53.6 ± 10.4φ	55.9 ± 14.0
LVmass/EDV ratio (g/mL)	1.29 ± 0.36	1.36 ± 0.48§	1.05 ± 0.22	1.30 ± 0.37

EDV: end-diastolic volume; EF: ejection fraction; LV: left ventricular.

* p = 0.0032;

# p = 0.0225 between baseline values of the two groups;

φ p = 0.0429 between baseline values of the two groups;

§ p = 0.015.

The LV mass showed a statistically significant 8.9% reduction in the TG (128.7 ± 38.9 g to 117.2 ± 27.2 g; p = 0.0032). Indexed LVmass (g/m^2^) showed a statistically significant reduction in the TG (p = 0.0032). An opposite trend occurred in the CG, but without statistical significance. Also, a similar LVmass/EDV ratio was present at baseline (TG = 1.29 ± 0.36 g/mL, and CG = 1.36 ± 0.48 g/mL; p = 0.63). At the end of the protocol, we observed a statistically significant reduction in the LVmass/EDV ratio in the TG (p = 0.015), with a value of 1.05 ± 0.22 g/mL. In the CG, the LVmass/EDV ratio had a final value of 1.30 ± 0.37 g/mL (p > 0.99).

## Discussion

The present study demonstrated that aerobic exercise training provided a positive LV remodeling, as evaluated by CMR, and modification of cardiovascular risk factors in a sample of male individuals after a first acute MI. The exercise training protocol was tailored to patients' needs after optimized pharmacological treatment to allow a widespread application, even to patients with residual ischemia, reproducing what happens in the "real world", where patients present inherent difficulties to adhere to a cardiac rehabilitation program.

### Aerobic capacity

There was a statistically significant increase of 38.7% in the peak oxygen uptake in the TG. This increase was associated with an increase in the peak power during exercise, as shown by the higher values of treadmill speed and inclination reached after the training period. Several studies have documented an increase in the peak oxygen uptake, from 10% to 46%, in post-MI patients undergoing a cardiac rehabilitation program,^12,14,15,17,19,23-25^ depending on the training intensity. There was also a statistically significant reduction in resting HR in the TG, an expression of the positive adaptation of the sinus node. It is also important to emphasize that the reduction in resting HR decreases the risk of cardiovascular events.^26^ Also, during aerobic exercise, it enables the increase of HR reserve from rest to maximum physical exercise.^27,28^ No favorable changes were found in the CG, reinforcing the favorable effects of the aerobic training protocol despite the use of ß-blockers.

### Left ventricular function, volume and mass

To control or inhibit cardiac remodeling is a treatment target for patients after MI. Many studies have shown that drugs like angiotensin-converting enzyme inhibitors, angiotensin receptor blockers, β-blockers and aldosterone antagonists present anti-remodeling properties.^29^ However, the achieved results are so far unsatisfactory.

Research continues in pharmacological and non-pharmacological interventions that can reverse and/or inhibit this process. A recent meta-analysis has demonstrated that even the time after a MI influences the results obtained.^30^

CMR has been extensively validated as a precise tool to measure volumes and masses in normal and pathological scenarios.^31^ It has also been demonstrated that small samples can be used to accurately determine mass and volume modifications following an intervention.^32^ No change in LVEF or LVEDV could be demonstrated in both groups after aerobic exercise training, which could reflect optimized pharmacological treatment.

In addition, LVmass decreased in TG and slightly increased in CG. Since both groups were similar regarding MI characteristics, it seems reasonable to raise the hypothesis that this opposite pattern could be attributed to the aerobic exercise intervention.

The same opposite pattern was demonstrated regarding LVmass/EDV, with better proportionality in the TG than in the CG, which developed eccentric remodeling. An evidence has demonstrated that LVmass/EDV ratio is close to 1 in children and adolescents.^33,34^ Our results suggest a trend toward reestablishment of a normal LVmass/EDV ratio in the TG. Another published study has identified different patterns of ventricular hypertrophy as indicators of worse prognosis in patients after MI in a 2-year follow-up.^35^

Our results do not confirm the negative effect of exercise on cardiac remodeling as observed by others.^8-10^ There is evidence in the literature that the benefits of exercise on cardiac function and remodeling after MI are due to distinct mechanisms: improved endothelial function, reduced systemic vascular resistance, reduced preload, adjustment in autonomic system, reduction in HR and blood pressure at rest and in submaximal loads, and reduction in the LV wall stress.^15,17,18,23,36^

Direct comparisons may be difficult because echocardiographic measurements for mass and function were done with 2D echocardiographic techniques, based on formulas and assumptions of the LV geometric shape.^37^ Fewer studies have used CMR for mass and function quantitation. Dubach et al.^19^ have studied 25 patients after MI with reduced LVEF (32.3 ± 6%). Twelve patients were randomized to perform high-intensity physical activity in a rehabilitation center. Patients in both groups underwent CMR evaluations initially and after 2 months. They observed a nonsignificant increase in LV end-systolic volume and LVEDV (2.5% and 4.8%, respectively) in TG, and no changes occurred in LVmass and EF in the groups. These same patients were followed up for 1 year, one group with vigorous physical activity, with a weekly energy expenditure of approximately 2,100 Kcal more than the CG. The cardiac volumes, mass and function measurements showed no significant difference, indicating that no deleterious effect of cardiac rehabilitation could be detected.^38^ Schmid et al.^39^ have evaluated 38 post-MI patients with an EF of 50.4 ± 12.7%, assigned either to combined endurance training and resistance training or to endurance training alone for 12 weeks. By CMR at the end of the training period, EF, stroke volume, LVEDV and end-systolic volume increased slightly in both groups. No deleterious effect on remodeling was observed.^39^

Finally, the medications were maintained during the IP in the present study, eliminating the possibility that the results may have been influenced by changes in medication doses.

### Study limitations

Our study has several limitations. The volunteers were not randomly assigned. For ethical reasons cardiac rehabilitation is offered to all MI patients in our institution as a standard of care. The CG participants did not accept to enroll in the rehabilitation program, but accepted to undergo the tests. This may be more close to the "real world" and allowed the completion of the protocol by all patients in the TG. On the other hand, we observed that the baseline LVmass was significantly different between the study groups. One possible explanation may be our selection method, but no other suitable way was available. Sample size is always a concern when dealing with continuous variables such as volumes and mass, but using CMR to quantify them seems to be appropriate because of its high reproducibility and accuracy.^28^ In addition, previous studies have indicated that, even in small samples, modifications due to interventions in structural and functional parameters can be detected.^29^

No specific infarct location was selected in order to provide a variety of conditions close to what is seen in clinical practice. Also, for logistical reasons, we have not performed images to quantify the extent of the scar area through the late enhancement on CMR. Such information could be useful in attempting to explain individual behavior in both groups.

Only male patients were included in our sample. This may have reduced the sample size, but may have assured that no gender related influence on the remodeling process would be present.^40^

The peak oxygen uptake was obtained indirectly through equations. Cardiopulmonary exercise analysis should be more appropriate for obtaining this variable. However, all TG patients showed an increase in treadmill speed and inclination.

The study showed the benefits of aerobic exercise training on functional capacity and cardiac function of patients after MI. However, the physiological mechanisms responsible for these changes were not evaluated.

## Conclusions

The present study showed a positive remodeling in the TG, as indicated by the slight increase in diastolic LV size associated with a reduction in LVmass. This was obtained with a moderate-intensity aerobic training that was effective in improving peak oxygen uptake and promoted benefic cardiovascular adaptations associated with a reduction in cardiovascular risk factors.
